# (*E*)-*N*′-(Furan-2-ylmethyl­ene)-4-(quinolin-8-yl­oxy)butanohydrazide

**DOI:** 10.1107/S1600536808032674

**Published:** 2008-10-15

**Authors:** Hai Xie, Shuang-Ming Meng, Yue-Qin Fan, Guo-Chen Yang

**Affiliations:** a College of Chemistry and Chemical Engineering, Shanxi Datong University, Datong, Shanxi 037009, People’s Republic of China

## Abstract

In the title mol­ecule, C_18_H_17_N_3_O_3_, the dihedral angle between the mean planes of the furan ring and the quinoline group is 77.4 (2)°. In the crystal structure, inter­molecular N—H⋯N hydrogen bonds link the mol­ecules into centrosymmetric dimers.

## Related literature

For general background, see: Cai *et al.* (2003 ▶); Chen *et al.* (2005 ▶); Park *et al.* (2006 ▶); Karmakar *et al.* (2007 ▶). For related structures, see: Zheng (2006 ▶); Zheng, Wu *et al.* (2006 ▶); Zheng, Li *et al.* (2006 ▶); Zheng *et al.* (2007 ▶, 2008 ▶).
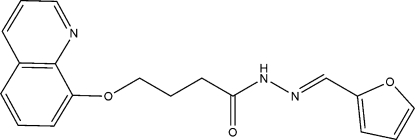


## Experimental

### 

#### Crystal data

C_18_H_17_N_3_O_3_
*M* *_r_* = 323.35
Triclinic, 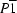

*a* = 8.2685 (17) Å
*b* = 8.6324 (17) Å
*c* = 12.765 (3) Å
α = 100.64 (3)°
β = 100.36 (4)°
γ = 109.50 (3)°
*V* = 814.9 (4) Å^3^
*Z* = 2
Mo *K*α radiation
μ = 0.09 mm^−1^
*T* = 295 K
0.33 × 0.26 × 0.21 mm

#### Data collection

Bruker SMART CCD area-detector diffractometer
Absorption correction: multi-scan (*SADABS*; Sheldrick, 1996 ▶) *T*_min_ = 0.970, *T*_max_ = 0.981
9399 measured reflections
2865 independent reflections
1632 reflections with *I* > 2σ(*I*)
*R*_int_ = 0.043

#### Refinement

*R*[*F* ^2^ > 2σ(*F* ^2^)] = 0.058
*wR*(*F* ^2^) = 0.193
*S* = 1.06
2865 reflections
218 parameters
21 restraints
H-atom parameters constrained
Δρ_max_ = 0.24 e Å^−3^
Δρ_min_ = −0.20 e Å^−3^

### 

Data collection: *SMART* (Bruker, 2007 ▶); cell refinement: *SAINT* (Bruker, 2007 ▶); data reduction: *SAINT*; program(s) used to solve structure: *SHELXS97* (Sheldrick, 2008 ▶); program(s) used to refine structure: *SHELXL97* (Sheldrick, 2008 ▶); molecular graphics: *SHELXTL* (Sheldrick, 2008 ▶); software used to prepare material for publication: *SHELXTL*.

## Supplementary Material

Crystal structure: contains datablocks global, I. DOI: 10.1107/S1600536808032674/lh2705sup1.cif

Structure factors: contains datablocks I. DOI: 10.1107/S1600536808032674/lh2705Isup2.hkl

Additional supplementary materials:  crystallographic information; 3D view; checkCIF report

## Figures and Tables

**Table 1:** Hydrogen-bond geometry (Å, °)

*D*—H⋯*A*	*D*—H	H⋯*A*	*D*⋯*A*	*D*—H⋯*A*
N2—H1⋯N1^i^	0.86	2.10	2.936 (4)	164

Symmetry code: (i) 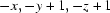.

**Fig. 1.**
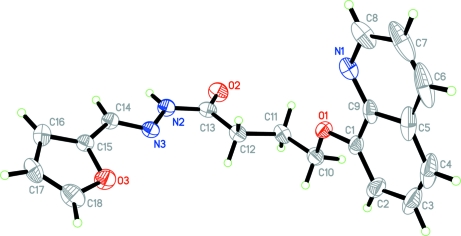
The molecular structure with displacement ellipsoids at the 30% probability level.

**Fig. 2.**
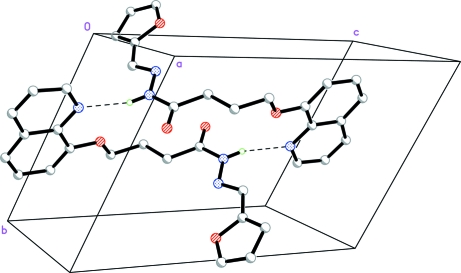
Part of the crystal structure showing hydrogen bonds as dashed lines. H atoms, except for those involved in hydrogen bonds, are not included.

## supplementary crystallographic information

### Comment

Synthsis of 8-Hydroxyquinoline and its derivatives have attracted a great interest due to their interesting biological activities and applications in coordination chemistry (Cai *et al.*, 2003; Chen *et al.*, 2005; Park *et al.*, 2006; Karmakar *et al.* 2007). Herein, we report the synthesis and crystal structure of the title compound, (I). The molecular structure of (I) is shown in Fig. 1. The conformation along the O1—C10—C11—C12—C13—N2—N3—C14 bond sequence is (-)*gauche*-*trans*-*trans*-(-)*gauche*-*trans*. The mean planes of the furan ring and quinoline group make a dihedral angle of 77.4 (2) °. In the crystal structure (Fig. 2), intermolecular N—H···N hydrogen bonds (Table 1) link the molecules into centrosymmetric dimers. Some crystal structures which are closely related to the title compound have already been studied (Zheng, 2006; Zheng, Wu *et al.*,2006; Zheng, *et al.*,2007; Zheng, Li *et al.*, 2006; Zheng, *et al.*, 2008.

### Experimental

Reagents and solvents used were of commercially available quality. The title complex (I) was synthesized according to the method of Zheng (2006). 4-(Quinolin-8-yloxy)butanohydrazide (0.01 mol), furan-2-carbaldehyde (0.01 mol), ethanol (40 ml) and some drops of acetic acid were added to a 100 ml flask and refluxed for 6 h. After cooling to room temperature, the solid product was separated by filtration. Colourless single crystals suitable for X-ray diffraction study were obtained by slow evaporation of a tetrahydrofuran solution over a period of 2 d.

### Refinement

All H atoms were placed in idealized positions (C—H = 0.93–0.97Å and N—H = 0.86Å) and refined as riding atoms with *U*_iso_(H) = 1.2U_eq_(C,*N*). In the molecule, some anisotropic displacemnt parameters of the atoms are larger than normal and restraints were applied in the form of the DELU and SIMU instructions in the SHELXL (Sheldrick, 2008) program.

### Crystal data

**Table:**

C_18_H_17_N_3_O_3_	*Z* = 2
*M**_r_* = 323.35	*F*(000) = 340
Triclinic, *P*1	*D*_x_ = 1.318 Mg m^−^^3^
Hall symbol: -P 1	Mo *K*α radiation, λ = 0.71073 Å
*a* = 8.2685 (17) Å	Cell parameters from 1197 reflections
*b* = 8.6324 (17) Å	θ = 2.6–20.5°
*c* = 12.765 (3) Å	µ = 0.09 mm^−^^1^
α = 100.64 (3)°	*T* = 295 K
β = 100.36 (4)°	Block, colorless
γ = 109.50 (3)°	0.33 × 0.26 × 0.21 mm
*V* = 814.9 (4) Å^3^	

### Data collection

**Table:**

Bruker SMART CCD area-detector diffractometer	2865 independent reflections
Radiation source: fine-focus sealed tube	1632 reflections with *I* > 2σ(*I*)
graphite	*R*_int_ = 0.043
φ and ω scans	θ_max_ = 25.0°, θ_min_ = 1.7°
Absorption correction: multi-scan (SADABS; Sheldrick, 1996)	*h* = −9→8
*T*_min_ = 0.970, *T*_max_ = 0.981	*k* = −10→10
9399 measured reflections	*l* = −15→15

### Refinement

**Table:**

Refinement on *F*^2^	Secondary atom site location: difference Fourier map
Least-squares matrix: full	Hydrogen site location: inferred from neighbouring sites
*R*[*F*^2^ > 2σ(*F*^2^)] = 0.058	H-atom parameters constrained
*wR*(*F*^2^) = 0.193	*w* = 1/[σ^2^(*F*_o_^2^) + (0.0865*P*)^2^ + 0.1859*P*] where *P* = (*F*_o_^2^ + 2*F*_c_^2^)/3
*S* = 1.06	(Δ/σ)_max_ < 0.001
2865 reflections	Δρ_max_ = 0.24 e Å^−^^3^
218 parameters	Δρ_min_ = −0.20 e Å^−^^3^
21 restraints	Extinction correction: SHELXL97 (Sheldrick, 2008), Fc^*^=kFc[1+0.001xFc^2^λ^3^/sin(2θ)]^-1/4^
Primary atom site location: structure-invariant direct methods	Extinction coefficient: 0.015 (6)

### Special details

**Table:**

Geometry. All e.s.d.'s (except the e.s.d. in the dihedral angle between two l.s. planes) are estimated using the full covariance matrix. The cell e.s.d.'s are taken into account individually in the estimation of e.s.d.'s in distances, angles and torsion angles; correlations between e.s.d.'s in cell parameters are only used when they are defined by crystal symmetry. An approximate (isotropic) treatment of cell e.s.d.'s is used for estimating e.s.d.'s involving l.s. planes.
Refinement. Refinement of *F*^2^ against ALL reflections. The weighted *R*-factor *wR* and goodness of fit *S* are based on *F*^2^, conventional *R*-factors *R* are based on *F*, with *F* set to zero for negative *F*^2^. The threshold expression of *F*^2^ > σ(*F*^2^) is used only for calculating *R*-factors(gt) *etc*. and is not relevant to the choice of reflections for refinement. *R*-factors based on *F*^2^ are statistically about twice as large as those based on *F*, and *R*- factors based on ALL data will be even larger.

### Fractional atomic coordinates and isotropic or equivalent isotropic displacement parameters (Å^2^)

**Table:**

	*x*	*y*	*z*	*U*_iso_*/*U*_eq_	
N1	0.2058 (3)	0.5842 (4)	0.8792 (2)	0.0700 (8)	
N2	−0.0972 (4)	0.3300 (3)	0.3273 (2)	0.0681 (8)	
H1	−0.1174	0.3754	0.2743	0.082*	
N3	−0.2378 (4)	0.2174 (3)	0.3521 (2)	0.0608 (7)	
O1	0.2645 (3)	0.3778 (2)	0.72254 (16)	0.0631 (6)	
O2	0.1947 (3)	0.4670 (3)	0.3574 (2)	0.0890 (8)	
C16	−0.7219 (5)	0.0732 (5)	0.2779 (3)	0.0811 (10)	
H16	−0.7644	0.1349	0.2353	0.097*	
C1	0.2775 (4)	0.3409 (4)	0.8220 (3)	0.0682 (9)	
C2	0.3186 (5)	0.2071 (5)	0.8455 (4)	0.1060 (15)	
H2	0.3394	0.1330	0.7916	0.127*	
C3	0.3287 (7)	0.1846 (8)	0.9529 (6)	0.147 (2)	
H3	0.3525	0.0920	0.9682	0.176*	
C4	0.3056 (7)	0.2905 (10)	1.0325 (5)	0.154 (3)	
H4	0.3176	0.2727	1.1026	0.184*	
C5	0.2631 (5)	0.4296 (8)	1.0135 (4)	0.1188 (17)	
C6	0.2347 (8)	0.5503 (12)	1.0944 (5)	0.164 (3)	
H6	0.2409	0.5392	1.1659	0.197*	
C7	0.1999 (8)	0.6759 (9)	1.0659 (4)	0.154 (3)	
H7	0.1858	0.7570	1.1185	0.185*	
C8	0.1837 (5)	0.6903 (6)	0.9582 (3)	0.1034 (15)	
H8	0.1556	0.7800	0.9411	0.124*	
C9	0.2483 (4)	0.4547 (5)	0.9052 (2)	0.0717 (10)	
C10	0.3224 (4)	0.2872 (4)	0.6405 (3)	0.0767 (10)	
H10A	0.2543	0.1657	0.6233	0.092*	
H10B	0.4470	0.3077	0.6677	0.092*	
C11	0.2946 (4)	0.3499 (4)	0.5394 (3)	0.0737 (10)	
H11A	0.3515	0.3051	0.4880	0.088*	
H11B	0.3517	0.4731	0.5600	0.088*	
C12	0.1001 (4)	0.2989 (4)	0.4818 (3)	0.0660 (9)	
H12A	0.0446	0.1756	0.4569	0.079*	
H12B	0.0413	0.3375	0.5344	0.079*	
C13	0.0731 (5)	0.3712 (4)	0.3847 (3)	0.0657 (9)	
C14	−0.3915 (4)	0.2060 (4)	0.3007 (2)	0.0617 (8)	
H14	−0.3984	0.2746	0.2532	0.074*	
C15	−0.5530 (4)	0.0908 (4)	0.3141 (2)	0.0591 (8)	
O3	−0.5432 (3)	−0.0227 (3)	0.3737 (2)	0.0872 (8)	
C18	−0.7117 (6)	−0.1128 (5)	0.3740 (4)	0.1000 (13)	
H18	−0.7433	−0.1998	0.4089	0.120*	
C17	−0.8239 (5)	−0.0625 (5)	0.3191 (3)	0.0867 (12)	
H17	−0.9466	−0.1055	0.3084	0.104*	

### Atomic displacement parameters (Å^2^)

**Table:**

	*U*^11^	*U*^22^	*U*^33^	*U*^12^	*U*^13^	*U*^23^
N1	0.0610 (17)	0.0779 (18)	0.0552 (16)	0.0069 (14)	0.0221 (13)	0.0102 (14)
N2	0.0650 (18)	0.0685 (17)	0.0741 (17)	0.0177 (14)	0.0275 (14)	0.0316 (14)
N3	0.0621 (17)	0.0560 (15)	0.0643 (15)	0.0150 (13)	0.0258 (13)	0.0206 (12)
O1	0.0712 (14)	0.0671 (13)	0.0592 (13)	0.0337 (11)	0.0168 (10)	0.0224 (10)
O2	0.0729 (17)	0.0872 (17)	0.1058 (19)	0.0133 (13)	0.0390 (14)	0.0396 (14)
C16	0.070 (2)	0.097 (3)	0.073 (2)	0.032 (2)	0.0116 (18)	0.020 (2)
C1	0.0464 (18)	0.068 (2)	0.077 (2)	0.0050 (15)	−0.0011 (15)	0.0366 (19)
C2	0.062 (2)	0.086 (3)	0.154 (4)	0.011 (2)	−0.011 (2)	0.065 (3)
C3	0.086 (3)	0.140 (4)	0.180 (5)	−0.006 (3)	−0.031 (4)	0.122 (4)
C4	0.081 (3)	0.199 (5)	0.126 (4)	−0.028 (3)	−0.024 (3)	0.119 (4)
C5	0.055 (2)	0.178 (4)	0.077 (3)	−0.024 (2)	−0.0051 (19)	0.079 (3)
C6	0.079 (4)	0.269 (8)	0.046 (3)	−0.051 (4)	0.011 (2)	0.031 (4)
C7	0.087 (4)	0.208 (7)	0.067 (4)	−0.039 (4)	0.036 (3)	−0.037 (4)
C8	0.078 (3)	0.109 (3)	0.082 (3)	−0.004 (2)	0.040 (2)	−0.015 (2)
C9	0.0455 (19)	0.092 (3)	0.0510 (19)	−0.0082 (17)	0.0031 (14)	0.0302 (19)
C10	0.061 (2)	0.072 (2)	0.088 (2)	0.0296 (18)	0.0090 (18)	0.0025 (19)
C11	0.058 (2)	0.082 (2)	0.069 (2)	0.0210 (17)	0.0216 (17)	−0.0016 (18)
C12	0.060 (2)	0.0655 (19)	0.069 (2)	0.0189 (16)	0.0242 (16)	0.0123 (16)
C13	0.071 (2)	0.0574 (19)	0.070 (2)	0.0195 (17)	0.0317 (18)	0.0178 (16)
C14	0.067 (2)	0.0621 (19)	0.0605 (18)	0.0215 (16)	0.0234 (16)	0.0245 (15)
C15	0.068 (2)	0.0585 (18)	0.0535 (17)	0.0218 (16)	0.0202 (15)	0.0199 (14)
O3	0.0827 (18)	0.0858 (17)	0.1002 (18)	0.0247 (14)	0.0323 (14)	0.0469 (14)
C18	0.083 (3)	0.092 (3)	0.113 (3)	0.004 (2)	0.046 (3)	0.034 (2)
C17	0.054 (2)	0.096 (3)	0.081 (2)	0.005 (2)	0.0213 (19)	−0.002 (2)

### Geometric parameters (Å, °)

**Table:**

N1—C8	1.314 (4)	C6—C7	1.306 (10)
N1—C9	1.357 (4)	C6—H6	0.9300
N2—C13	1.358 (4)	C7—C8	1.389 (7)
N2—N3	1.374 (3)	C7—H7	0.9300
N2—H1	0.8600	C8—H8	0.9300
N3—C14	1.283 (4)	C10—C11	1.499 (5)
O1—C1	1.360 (4)	C10—H10A	0.9700
O1—C10	1.437 (4)	C10—H10B	0.9700
O2—C13	1.222 (4)	C11—C12	1.517 (4)
C16—C15	1.337 (5)	C11—H11A	0.9700
C16—C17	1.445 (5)	C11—H11B	0.9700
C16—H16	0.9300	C12—C13	1.501 (4)
C1—C2	1.375 (5)	C12—H12A	0.9700
C1—C9	1.419 (5)	C12—H12B	0.9700
C2—C3	1.412 (7)	C14—C15	1.435 (4)
C2—H2	0.9300	C14—H14	0.9300
C3—C4	1.322 (9)	C15—O3	1.361 (4)
C3—H3	0.9300	O3—C18	1.351 (4)
C4—C5	1.408 (9)	C18—C17	1.300 (5)
C4—H4	0.9300	C18—H18	0.9300
C5—C9	1.428 (5)	C17—H17	0.9300
C5—C6	1.436 (9)		
			
C8—N1—C9	117.8 (3)	C1—C9—C5	119.4 (4)
C13—N2—N3	121.3 (3)	O1—C10—C11	107.8 (3)
C13—N2—H1	119.3	O1—C10—H10A	110.2
N3—N2—H1	119.3	C11—C10—H10A	110.2
C14—N3—N2	114.7 (3)	O1—C10—H10B	110.2
C1—O1—C10	118.0 (3)	C11—C10—H10B	110.2
C15—C16—C17	105.0 (3)	H10A—C10—H10B	108.5
C15—C16—H16	127.5	C10—C11—C12	113.4 (3)
C17—C16—H16	127.5	C10—C11—H11A	108.9
O1—C1—C2	124.8 (4)	C12—C11—H11A	108.9
O1—C1—C9	115.0 (3)	C10—C11—H11B	108.9
C2—C1—C9	120.2 (4)	C12—C11—H11B	108.9
C1—C2—C3	118.7 (5)	H11A—C11—H11B	107.7
C1—C2—H2	120.7	C13—C12—C11	113.1 (3)
C3—C2—H2	120.7	C13—C12—H12A	109.0
C4—C3—C2	122.3 (6)	C11—C12—H12A	109.0
C4—C3—H3	118.8	C13—C12—H12B	109.0
C2—C3—H3	118.8	C11—C12—H12B	109.0
C3—C4—C5	121.5 (6)	H12A—C12—H12B	107.8
C3—C4—H4	119.2	O2—C13—N2	119.3 (3)
C5—C4—H4	119.2	O2—C13—C12	123.6 (3)
C4—C5—C9	117.7 (6)	N2—C13—C12	117.1 (3)
C4—C5—C6	125.4 (5)	N3—C14—C15	122.2 (3)
C9—C5—C6	116.8 (6)	N3—C14—H14	118.9
C7—C6—C5	118.9 (6)	C15—C14—H14	118.9
C7—C6—H6	120.5	C16—C15—O3	110.4 (3)
C5—C6—H6	120.5	C16—C15—C14	130.9 (3)
C6—C7—C8	121.1 (7)	O3—C15—C14	118.7 (3)
C6—C7—H7	119.5	C18—O3—C15	106.4 (3)
C8—C7—H7	119.5	C17—C18—O3	111.3 (4)
N1—C8—C7	123.6 (5)	C17—C18—H18	124.4
N1—C8—H8	118.2	O3—C18—H18	124.4
C7—C8—H8	118.2	C18—C17—C16	106.9 (3)
N1—C9—C1	118.9 (3)	C18—C17—H17	126.5
N1—C9—C5	121.7 (4)	C16—C17—H17	126.5
			
C13—N2—N3—C14	171.9 (3)	C6—C5—C9—N1	−1.4 (5)
C10—O1—C1—C2	−9.5 (4)	C4—C5—C9—C1	−0.4 (5)
C10—O1—C1—C9	169.3 (2)	C6—C5—C9—C1	179.1 (4)
O1—C1—C2—C3	179.6 (3)	C1—O1—C10—C11	179.9 (2)
C9—C1—C2—C3	0.8 (5)	O1—C10—C11—C12	−68.5 (3)
C1—C2—C3—C4	−2.2 (7)	C10—C11—C12—C13	176.4 (3)
C2—C3—C4—C5	2.2 (9)	N3—N2—C13—O2	177.3 (3)
C3—C4—C5—C9	−0.9 (7)	N3—N2—C13—C12	−4.6 (4)
C3—C4—C5—C6	179.6 (5)	C11—C12—C13—O2	−2.9 (4)
C4—C5—C6—C7	178.5 (5)	C11—C12—C13—N2	179.1 (3)
C9—C5—C6—C7	−1.0 (8)	N2—N3—C14—C15	178.1 (3)
C5—C6—C7—C8	2.4 (10)	C17—C16—C15—O3	0.2 (4)
C9—N1—C8—C7	−0.6 (5)	C17—C16—C15—C14	−179.2 (3)
C6—C7—C8—N1	−1.7 (8)	N3—C14—C15—C16	172.0 (3)
C8—N1—C9—C1	−178.4 (3)	N3—C14—C15—O3	−7.3 (4)
C8—N1—C9—C5	2.1 (4)	C16—C15—O3—C18	0.0 (4)
O1—C1—C9—N1	2.0 (4)	C14—C15—O3—C18	179.5 (3)
C2—C1—C9—N1	−179.2 (3)	C15—O3—C18—C17	−0.2 (4)
O1—C1—C9—C5	−178.5 (3)	O3—C18—C17—C16	0.3 (5)
C2—C1—C9—C5	0.4 (4)	C15—C16—C17—C18	−0.2 (4)
C4—C5—C9—N1	179.2 (3)		

### Hydrogen-bond geometry (Å, °)

**Table:**

*D*—H···*A*	*D*—H	H···*A*	*D*···*A*	*D*—H···*A*
N2—H1···N1^i^	0.86	2.10	2.936 (4)	164

Symmetry codes: (i) −*x*, −*y*+1, −*z*+1.
